# Violence across the lifespan and its association with depression: cumulative effects and psychosocial moderation in the UK Biobank

**DOI:** 10.1007/s00127-026-03079-3

**Published:** 2026-04-29

**Authors:** Madison L Cassady, Xuqian Li, Lena K. L. Oestreich

**Affiliations:** 1 School of Psychological Sciences, Monash University, Melbourne, Australia; 2 Australian Institute for Bioengineering and Nanotechnology (AIBN), The University of Queensland, Brisbane, Australia; 3 School of Psychology, The University of Queensland, Brisbane, Australia

**Keywords:** Depression, Childhood maltreatment, Lifetime violence, Intimate partner violence (IPV), UK Biobank, Major depressive disorder (MDD)

## Abstract

**Purpose:**

Violence exposure is a well-established risk factor for depression, yet few large-scale studies have examined how distinct violence types across the lifespan interact to shape depression trajectories or how psychosocial factors may moderate these effects.

**Methods:**

Drawing on data from 35,921 UK Biobank participants, we examined cross-sectional and prospective associations of childhood maltreatment, lifetime violence, and intimate partner violence (IPV) with depressive symptom severity and depression diagnosis (case-control design with propensity score matching). Hierarchical regression models tested main effects adjusting for confounders (age, sex, socioeconomic status [SES]), and interaction terms tested moderation by SES, social support, and loneliness.

**Results:**

All violence exposures were independently associated with greater depression severity and higher odds of depression diagnosis. Cumulative exposure demonstrated additive, synergistic, and nonlinear effects, with co-occurring violence types compounding psychological risk. Loneliness amplified violence-depression associations, while social support buffered these relationships. SES did not moderate violence effects. Prospective analyses confirmed that violence exposure predicted future depressive symptoms beyond baseline severity. In case-control analyses of clinical depression, IPV contributed the highest population attributable fraction (1.8%), followed by lifetime violence (1.1%) and childhood maltreatment (0.6%).

**Conclusions:**

Depression risk following violence is shaped not only by violence exposure per se, but also by modifiable psychosocial factors, with loneliness and social support emerging as critical intervention targets. These findings highlight the need for integrated violence screening and targeted psychosocial interventions within mental health and public health strategies.

**Supplementary Information:**

The online version contains supplementary material available at https://doi.org/10.1007/s00127-026-03079-3.

## Introduction

Depression represents one of the leading causes of disability worldwide, with a complex etiology involving both genetic and environmental factors [1]. Among environmental contributors, exposure to violence across the lifespan is a well-established risk factor for the development and persistence of depressive symptoms [2, 3]. Although substantial evidence links specific forms of violence to adverse mental health outcomes, less is known about how different types of violence exposure interact or how psychosocial factors may modify these relationships over time.

Violence exposure can occur in multiple contexts: during childhood as maltreatment (specifically physical, sexual, or emotional abuse), throughout life in the form of community or interpersonal violence, and within romantic relationships as intimate partner violence (IPV) [4, 5]. Childhood maltreatment, defined here as exposure to emotional, physical, or sexual abuse during childhood, has been robustly associated with disrupted developmental trajectories and heightened susceptibility to depression later in life [6]. Similarly, IPV, which includes emotional, physical, and sexual abuse by a partner, has been linked to depressive morbidity [7]. Beyond IPV, lifetime exposures to violence from non-partners, such as sexual assault, violent crime victimization, or witnessing violent death, are associated with persistent depressive symptoms, functional impairment, and elevated healthcare burden [8]. Importantly, these experiences often co-occur, and cumulative exposure to multiple violence types may confer compounded psychological risks [3]. Research has demonstrated that childhood maltreatment increases vulnerability to revictimization in adulthood, potentially creating cascading patterns of risk [9]. However, many studies have examined isolated exposures to violence or relied on composite measures, obscuring the distinct contributions and interactions between different forms of violence.

The diathesis-stress model posits that adverse experiences, such as childhood maltreatment and violence exposure interact with individual vulnerability factors to precipitate depressive outcomes [10]. Within this framework, psychosocial factors, such as social support, socioeconomic status (SES) and loneliness, may play critical roles in shaping mental health trajectories. These factors can exert direct effects on depression risk or act as moderators, either exacerbating vulnerability (e.g., loneliness) or buffering individuals against the psychological impact of trauma (e.g., social support) [11–13]. SES is not only a social determinant of health but also a potential psychological moderator, with lower SES linked to both heightened exposure to adversity and diminished coping resources [14]. Loneliness, likewise has been reported as a potent and independent predictor of depressive outcomes and may amplify the negative consequences of early or ongoing trauma [6]. Conversely, perceived social support has been shown to mitigate the risk conferred by violence exposure, acting as a protective mechanism [15]. Nevertheless, it remains unclear whether these factors merely exert additive effects alongside violence exposure or actively moderate its impact on depression.

Previous studies have been constrained by sample sizes insufficient to detect interaction effects with psychosocial factors or to examine complex patterns of co-occurring violence exposures [16]. Moreover, few studies have simultaneously examined both continuous measures of depressive symptom severity in the general population and clinical diagnostic outcomes, thereby limiting our understanding of how violence exposure impacts the full spectrum of depressive psychopathology [17]. Large-scale, population-based resources such as the UK Biobank offer unique opportunities to disentangle the multifaceted relationships between violence exposure, psychosocial context, and mental health outcomes.

Drawing on longitudinal data from over 35,000 participants, the present study systematically examines how childhood maltreatment, lifetime violence, and IPV contribute to both depressive symptom severity and clinical depression diagnosis. We further test whether psychosocial factors modify these associations and explore additive, interactive, and non-linear cumulative effects of violence exposure. This research aims to elucidate pathways linking violence to depression and to identify modifiable psychosocial targets for prevention and intervention. Building on previous literature, we hypothesised that each form of violence would independently predict elevated depression risk, with cumulative exposure associated with disproportionately higher symptom severity. Furthermore, we expected that psychosocial factors would exert both independent and moderating effects on depression. Finally, we anticipated that violence exposures and psychosocial factors would prospectively predict future depressive symptoms even after accounting for initial symptom severity, highlighting the enduring mental health burden of violence across the lifespan.

## Methods

### Study design and participants

The UK Biobank is a large, population-based longitudinal cohort study that recruited over 500,000 participants aged 40–69 years from across the United Kingdom [18]. Data for the present study were primarily drawn from two main assessment periods. The initial assessment (T1) occurred during the 2016–2017 Mental Health Questionnaire (MHQ), where participants completed online questionnaires assessing depressive symptoms, childhood adversity, lifetime violence exposure, and IPV [19]. The follow-up assessment (T2) of depressive symptoms was conducted through the 2019–2021 Mental Wellbeing Follow-up Questionnaire, administered approximately 2–4 years later [20]. For a detailed overview of the UK biobank data, see [18].

The study was approved by the NHS National Research Ethics Service (11/NW/0382) and was ratified by the University of Queensland (2023/HE000221). All participants gave full informed written consent.

### Measures

Depressive symptoms were assessed using nine items that correspond to the diagnostic criteria in the Patient Health Questionnaire (PHQ-9), a validated and reliable (*α* = 0.89) screening tool for depression [21]. These items (Field IDs: 20518, 20510, 20507, 20519, 20514, 20511, 20513, 20508, 20517) assessed symptoms at the initial assessment (T1), with equivalent measures (Field IDs: 29009, 29003, 29007, 29005, 29002, 29006, 29010, 29008, 29004) at follow-up (T2). A summed score was calculated, with higher values indicating greater symptom severity. A binary depression diagnosis variable was derived from three sources: ICD-9 diagnosis codes (Field ID: 41271), ICD-10 diagnosis codes (Field ID: 41270), and self-reported physician diagnosis of depression (Field ID: 29000). Participants meeting any of these criteria were classified as having a depression diagnosis.

Childhood maltreatment was assessed using four validated items from the Childhood Trauma Screener [22]: feeling loved as a child (Field ID: 20489), physical abuse (Field ID: 20488), feeling hated (Field ID: 20487), and sexual abuse (Field ID: 20490). A childhood emotional maltreatment variable was created by combining the “feeling hated” item with the reverse-scored “feeling loved” item, reflecting perceived emotional rejection and lack of emotional safety within the caregiving environment. A composite childhood maltreatment index was created by standardizing and summing physical, sexual, and emotional abuse scores. While maltreatment can include neglect [23], the current study focused on the violent exposure dimensions of physical, sexual, and emotional abuse. Lifetime violence exposure from non-partners was assessed through items measuring sexual assault (Field ID: 20531), violent crime victimization (Field ID: 20529), and witnessed violent death (Field ID: 20530). IPV was assessed through items measuring lack of emotional support (reverse-scored confiding in partner; Field ID: 20522), physical violence (Field ID: 20523), belittlement/emotional abuse (Field ID: 20521), and sexual violence (Field ID: 20524). Childhood maltreatment and IPV items were rated on a 5-point scale (0 = *Never true* to 4 = *Very often true*). Lifetime violence items were rated on a 3-point scale (0 = *Never*, 1 = *Yes*,* but not in the last 12 months*, 2 = *Yes*,* within the last 12 months*). Given these differing response formats, individual items were z-scored prior to summation to ensure equal weighting within each composite index. Equal weighting was chosen because each item represents a distinct form of adversity considered equally relevant to cumulative violence exposure, consistent with cumulative risk frameworks in developmental psychopathology [24]. Of note, timeframes differ across violence measures, reflecting the original UK Biobank design: childhood maltreatment items assessed experiences during childhood retrospectively, lifetime violence items distinguished between recent (past 12 months) and lifetime occurrence, and IPV items assessed experiences within intimate relationships without a specific timeframe. We furthermore acknowledge potential overlap between variables; for example, childhood sexual abuse could also be reported as lifetime sexual assault. The simultaneous modelling approach estimates unique contributions of each violence type while controlling for shared variance.

SES was measured using household income (Field ID: 738) and educational attainment (Field ID: 6138). A composite SES index was created by standardizing and summing these variables. Although these indicators are partially distinct, both contribute to overall socioeconomic position and access to resources relevant for mental health. Composite SES measures have been shown to capture social gradients in health more robustly than single indicators [25] and our aim was to adjust for broad socioeconomic disadvantage rather than to examine specific mechanisms of education or income effects. Social support was assessed through satisfaction with friendships (Field ID: 4570), satisfaction with family relationships (Field ID: 4559), frequency of social activities (Field ID: 6160), and ability to confide in someone (Field ID: 2110). A social support index was created by standardizing and adding these measures. Loneliness was assessed with the item often feeling lonely (Field ID: 2020). Age at recruitment (Field ID: 21022) and sex (Field ID: 31) were included as covariates in all analyses. For a detailed description of the UK Biobank Field IDs, response options, original coding, recoding procedures, and variable construction, see Supplementary Table 1.

**Table 1:** UK Biobank Descriptive Statistics

Variable	Total Sample (*N* = 36,378)	Depression Group (*N* = 7,295)	Matched Controls (*N* = 7,295)
**Age (years)**	56 ± 7.6	54.5 ± 7.6	54.5 ± 7.6
**Sex**			
Female	20,417 (56.1%)	4,951 (67.9%)	4,951 (67.9%)
Male	15,961 (43.9%)	2,344 (32.3%)	2,344 (32.3%)
**Ethnicity** ^**a**^			
White	34,585 (96.3%)	6,852 (97.2%)	6,720 (95.3%)
Asian	499 (1.4%)	49 (0.7%)	136 (1.9%)
Black	316 (0.9%)	47 (0.7%)	75 (1.1%)
Mixed	217 (0.6%)	53 (0.8%)	47 (0.7%)
Other	216 (0.6%)	33 (0.5%)	60 (0.9%)
Unknown	88 (0.2%)	18 (0.3%)	14 (0.2%)
**Education**			
College or University degree	18,576 (51.1%)	3,615 (49.6%)	3,801 (52.1%)
High School diploma	5,161 (14.2%)	1,080 (14.8%)	1,107 (15.2%)
Secondary education	6,853 (18.8%)	1,465 (20.1%)	1,338 (18.3%)
Basic Secondary education	1,160 (3.2%)	274 (3.8%)	235 (3.2%)
Vocational post-secondary education	1,495 (4.1%)	261 (3.6%)	267 (3.7%)
Prefer not to answer	3,133 (8.6%)	600 (8.2%)	547 (7.5%)
**Income**			
Less than 18,000	4,460 (12.3%)	1,215 (16.7%)	735 (10.1%)
18,000 to 30,999	8,149 (22.4%)	1,692 (23.2%)	1,563 (21.4%)
31,000 to 51,999	10,532 (29%)	2,136 (29.3%)	2,095 (28.7%)
52,000 to 100,000	9,870 (27.1%)	1,787 (24.5%)	2,162 (29.6%)
Greater than 100,000	3,367 (9.3%)	465 (6.4%)	740 (10.1%)
**Employment**			
In paid employment/self-employed	23,266 (64.0%)	4,799 (65.8%)	5,041 (69.1%)
Retired	10,778 (29.6%)	1,805 (24.7%)	1,725 (23.6%)
Looking after home/family	960 (2.6%)	246 (3.4%)	253 (3.5%)
Unable to work (sickness/disability)	375 (1.0%)	203 (2.8%)	56 (0.8%)
Unemployed	499 (1.4%)	120 (1.6%)	101 (1.4%)
Doing unpaid/voluntary work	220 (0.6%)	41 (0.6%)	55 (0.8%)
Full or part-time student	101 (0.3%)	36 (0.5%)	24 (0.3%)
Prefer not to answer/None of above	179 (0.5%)	45 (0.6%)	40 (0.5%)

Continuous variables are presented as Mean ± SD; categorical variables are presented as n (%)

^a^Ethnicity was summarized using the following category labels: White (British, Irish, Any other white background), Mixed (White and Black Caribbean, White and Black African, White and Asian, Any other mixed background), Black (Caribbean, African, Any other Black background), Asian (Indian, Pakistani, Bangladeshi, Any other Asian background, Chinese)

### Data analysis

Complete case analysis was used, i.e. participants with missing data on any study variable were excluded. To assess potential selection bias, we compared included participants with those excluded due to missing data among MHQ completers on demographic and study variables.

Hierarchical multiple regression analyses were conducted to examine associations between three forms of violence exposure (childhood maltreatment, lifetime violence, IPV) and depressive symptom severity at T1 (PHQ-9 scores). In a first step, violence exposures were entered as predictors, adjusting for confounders (age, age squared, sex and SES). In a second step, social support and loneliness were added to examine whether these factors explained additional variance in depressive symptom severity. Sensitivity analyses additionally adjusted for parental history of depression (maternal and paternal). To test moderation, we specified three separate models with interaction terms between each violence type and SES, social support and loneliness. All continuous predictors were mean-centered before computing interactions. To assess whether simultaneous modelling attenuated violence effects due to shared variance or potential mediation, we also fit separate models for each violence type. Lastly, to examine the independent contribution of individual items on depression outcomes, we conducted supplementary item-level analyses. This approach was taken to assess whether composite associations were driven by specific maltreatment subtypes and to provide transparency regarding the relative contribution of items with differing endorsement rates.

Cumulative violence exposure effects were assessed in three ways: (1) a linear model using a summed violence exposure index, (2) a categorical model grouping participants by number of violence types experienced (one, two, or three types), and (3) a nonlinear model with a squared term to test for accelerating effects. To explore synergistic effects beyond their independent contributions, we constructed an interaction model including all pairwise interactions between the three violence types. Longitudinal models were used to predict depressive symptom severity at T2 from T1 violence exposures. We report two models: (1) adjusting for confounders only (age, sex, SES), which estimates the total prospective effect of violence on T2 depression, and (2) additionally adjusting for T1 depression, which examines whether violence predicts residualized change in depression symptoms.

Secondary analyses used a matched case-control design. Participants with a depression diagnosis were matched 1:1 to undiagnosed controls on age and sex using nearest neighbour propensity score matching (caliper = 0.2). Logistic regression models estimated associations between violence exposures and depression diagnosis, first with violence predictors and SES, and then with additional inclusion of social support and loneliness. Sensitivity analyses additionally adjusted for parental history of depression. Interaction terms were used to test moderation by psychosocial variables. Separate models and item-level analyses, as described above, were also conducted for depression diagnosis. Cumulative violence exposure effects on depression diagnosis were analysed with linear, categorical, and quadratic models. Population attributable fractions (PAFs) were estimated to quantify the proportion of depression diagnoses associated with violence exposures, assuming a causal relationship. PAFs were calculated from models adjusting for confounders (age, sex, SES).

All models controlled for age, age^2^, sex and SES. Multicollinearity was assessed using variance inflation factors (VIFs), with acceptable values between 1 and 5. Model fit was evaluated using adjusted *R²* and AIC. All analyses were conducted using RStudio (Version 2024.12.1) [26]. A total of 16 regression models were tested. To correct for multiple comparisons, a Bonferroni-adjusted alpha of 0.0031 (0.05 / 16) was applied. The code for the data analysis is publicly available here: https://github.com/LenaOestreich13/UKB-Violence-Depression.

## Results

**Fig. 1:**
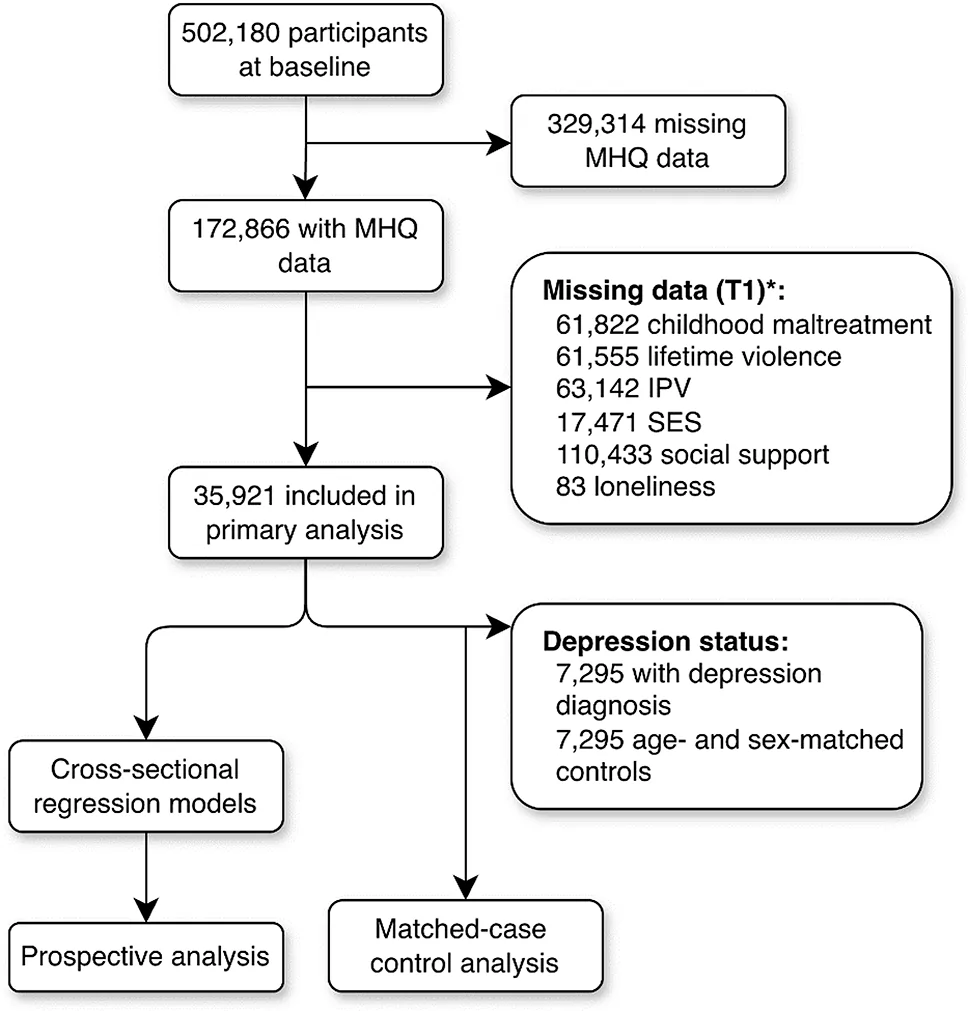
Participant flow diagram for the primary analysis and matched case-control sample. Flowchart illustrating the selection of participants from the UK Biobank dataset. *Categories overlap, i.e. participants could have missing data on multiple variables

After excluding participants with incomplete or missing data, 35,921 individuals were included in the analyses (see Fig. 1). Comparison of included and excluded participants indicated no sampling bias, revealing similar levels of violence exposure, SES, loneliness, and social support across groups (Supplementary Table 2). However, included participants were slightly older and had higher baseline depression severity. The final included participants ranged in age from 40 to 70 years (*M* = 56.1, *SD* = 7.6). Of the sample, 56.1% identified as female and 43.9% as male. Table 1 presents the demographic characteristics of the study sample. While the full range of PHQ-9 scores (0–27) was represented, the average depression severity score was relatively low (*M* = 2.65, *SD* = 3.57). Individual depressive symptom scores, estimates of violence exposure, and psychosocial factors are summarised in Supplementary Tables 3–5.

Internal consistency for the violence indices varied: childhood maltreatment (*α* = 0.63) and IPV (*α* = 0.60) showed acceptable reliability, while the lifetime violence index was lower (*α* = 0.22). The lower alpha for lifetime violence reflects that items capture conceptually distinct experiences (sexual assault, violent crime, witnessed violent death) that do not necessarily co-occur; this index represents cumulative exposure breadth rather than a unidimensional construct. Social support showed modest internal consistency (*α* = 0.48), reflecting the breadth of support domains assessed. Income and education, combined to form the SES composite, showed a modest positive correlation (*r* = 0.15), consistent with these variables capturing partially distinct dimensions of socioeconomic position.

### Associations of maltreatment, violence exposures and psychosocial factors on depression severity

**Fig. 2:**
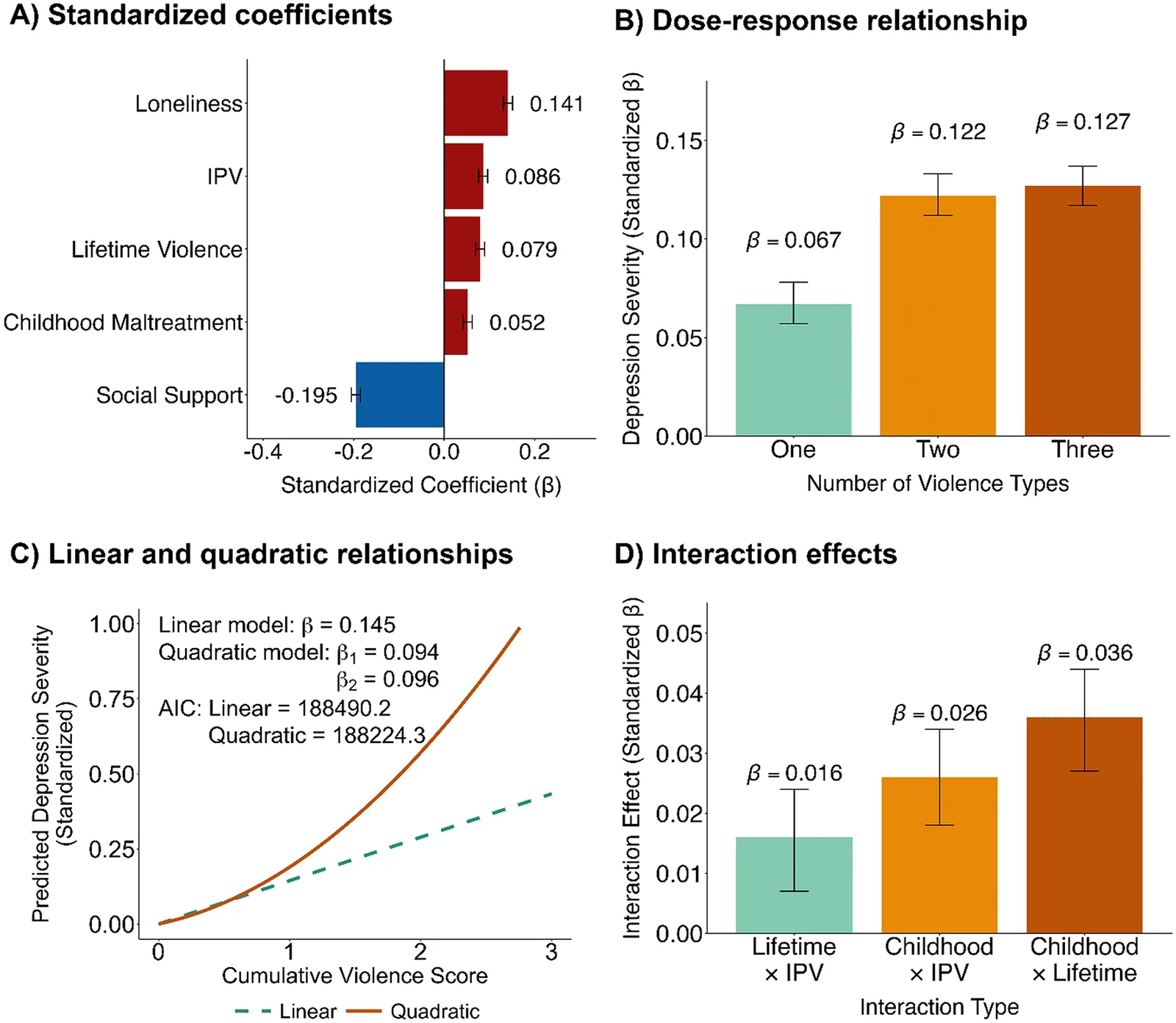
Associations between violence exposures, psychosocial factors, and depressive symptom severity. (**A**) Standardized regression coefficients showing independent associations of loneliness, intimate partner violence (IPV), lifetime violence, childhood maltreatment, socioeconomic status (SES), and social support with depression severity. (**B**) Dose-response relationship: depression severity increases with exposure to a greater number of violence types (one, two, or three). (**C**) Cumulative violence score predicting depression severity, showing better fit for a quadratic (accelerating) model compared to a linear model. (**D**) Interaction effects between different violence types indicating synergistic risk for greater depression severity. Error bars represent 95% confidence intervals.

In cross-sectional analyses predicting T1 depression severity, VIFs for all models ranged from 1.05 to 1.1, indicating low multicollinearity, with the exception of age and age², which showed high multicollinearity (VIFs > 200) due to their mathematical dependence. In the initial model, childhood maltreatment (*β* = 0.046, *p* < 0.001), lifetime violence (*β* = 0.099, *p* < 0.001), and IPV (*β* = 0.074, *p* < 0.001) were significant independent predictors of depressive severity. The model explained 6.1% of the variance in depression severity, *F*(7, 35,913) = 334.1, ${R}_{adj}^{2}$ = 0.061, *p* < 0.001. Sensitivity analyses additionally adjusting for parental history of depression (maternal and paternal) yielded consistent results (childhood maltreatment: *β* = 0.041, *p* < 0.001; lifetime violence: *β* = 0.096, *p* < 0.001; IPV: *β* = 0.072, *p* < 0.001). Adding social support and loneliness in a second step significantly improved model fit, *△**R*^*2*^ = 0.067, *F*_change_(2, 35,911) = 1377.1, *p <* 0.001. The full model explained 12.8% of the variance in depression severity, *F*(9,35,911) = 585.8, ${R}_{adj}^{2}$ = 0.128, *p* < 0.001. Limited social support emerged as the strongest predictor (*β* = -0.195, *p* < 0.001), followed closely by loneliness (*β* = 0.141, *p* < 0.001) (see Fig. 2A).

When each violence type was modelled separately, effect sizes were larger than in the simultaneous model: childhood maltreatment (*β* = 0.077, *p* < 0.001), lifetime violence (*β* = 0.12, *p* < 0.001), and IPV (*β* = 0.097, *p* < 0.001). The attenuation in the simultaneous model is consistent with shared variance among correlated exposures and potential mediation pathways (e.g., childhood maltreatment increasing risk for later IPV). Item-level analyses (Supplementary Table 6) indicated that emotional forms of maltreatment were the strongest individual predictors of depression severity: IPV-related belittlement (*β* = 0.144, *p* < 0.001) and childhood ‘felt hated’ (*β* = 0.083, *p* < 0.001) showed the largest independent associations. In contrast, physical IPV was the only non-significant predictor when controlling for other exposures.

SES significantly moderated the relationship between lifetime violence and depression (*β* = -0.014, *p* = 0.005), suggesting that higher SES may buffer the negative impact of lifetime violence on depression symptoms. However, SES did not significantly moderate the associations between childhood maltreatment or IPV and depression, indicating its buffering role may be specific to lifetime violence exposure. In contrast, social support significantly moderated the associations between all three forms of violence and depression: childhood maltreatment × social support (*β* = -0.015, *p* = 0.005), lifetime violence × social support (*β* = -0.027, *p* < 0.001), and IPV × social support (*β* = -0.02, *p* < 0.001), indicating that greater social support buffered the psychological impact of violence. Conversely, loneliness significantly amplified the effects of childhood maltreatment (*β* = 0.026, *p* < 0.001) and lifetime violence (*β* = 0.022, *p* < 0.001) on depression severity. However, its interaction with IPV was not statistically significant, suggesting loneliness does not exacerbate the impact of IPV on depression.

### Cumulative and nonlinear effects of violence on depression

We next tested whether cumulative violence exposure demonstrated additive or nonlinear associations with depression severity, while covarying for age, age ^2^, sex, SES, social support, and loneliness. A categorical model revealed a clear dose-response pattern, *F*(3, 35,911) = 333.68, *p* < 0.001: compared to individuals with no violence exposure, those exposed to one (*β* = 0.067, *p* < 0.001), two (*β* = 0.122, *p* < 0.001), and three types of violence (*β* = 0.127, *p* < 0.001; see Fig. 2B) showed progressively greater depression severity. This model explained 13.1% of the variance in depression severity, *F*(10, 35,910) = 603.3, ${R}_{adj}^{2}$= 0.131, *p* < 0.001, and demonstrated better fit (*AIC* = 188,334.8) than the linear model (*AIC* = 188,490.2), supporting a stepwise additive effect. A nonlinear model including both linear and quadratic terms for cumulative violence exposure showed the best overall fit (*AIC* = 188,224.3). The quadratic term was significant (*β* = 0.096, *p* < 0.001), indicating that depression severity increased at an accelerating rate with exposure to more violence types (see Fig. 2C). This model explained slightly more variance (13.4%) than the linear and categorical models, *F*(10, 35,910) = 694.4, ${R}_{adj}^{2}$= 0.134, *p* < 0.001.

We also tested whether interactions between violence types yielded synergistic effects. All pairwise combinations were significant: childhood maltreatment × lifetime violence (*β* = 0.036, *p* < 0.001), childhood maltreatment × IPV (*β* = 0.026, *p* < 0.001), and lifetime violence × IPV (*β* = 0.016, *p* = 0.004; see Fig. 2D). These findings indicate that experiencing multiple types of violence has a compounded effect on depression severity, beyond the sum of their individual contributions (*AIC* = 188,364.2; see Supplementary Fig. 1). Together, the models provide robust evidence that cumulative and co-occurring violence exposures significantly exacerbate depression severity. The strongest effects were observed among individuals with multiple forms of violence, and nonlinear patterns suggest that risk accelerates at higher exposure levels.

### Prospective Associations Between Violence Exposure and Depression

**Fig. 3:**
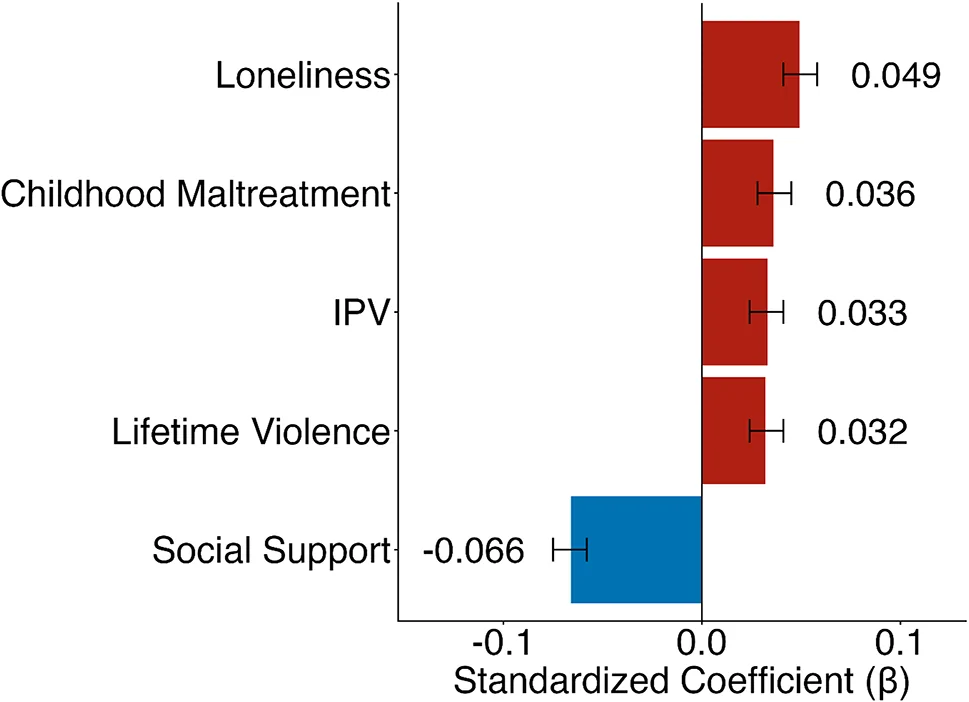
Prospective predictors of depressive symptom severity at follow-up. Standardized coefficients from longitudinal regression analysis predicting depressive symptoms at Time 2 from baseline violence exposures (childhood maltreatment, lifetime violence, intimate partner violence (IPV)) and psychosocial factors (socioeconomic status (SES), social support, loneliness), controlling for baseline depression severity. Loneliness and social support remain significant prospective predictors alongside violence exposures.

Depression severity was significantly increased at T2 (*range* = 0–27, *M* = 2.72, *SD* = 3.71) relative to T1: *t*(35,920) = -4.28, *p* < 0.001, *d* = 0.02. We conducted a prospective regression model to assess whether violence exposure at T1 predicted depression severity at T2. In the model adjusting for confounders only (age, age^2^, sex, SES), all violence types significantly predicted T2 depression: childhood maltreatment (*β* = 0.060, *p* < 0.001), lifetime violence (*β* = 0.095, *p* < 0.001), and IPV (*β* = 0.071, *p* < 0.001). This model explained 5.2% of the variance in T2 depression, *F*(7, 35,913) = 279.1, *R²adj* = 0.051, *p* < 0.001. When additionally adjusting for T1 depression to examine residualized change, violence effects were attenuated but remained significant: childhood maltreatment (*β* = 0.033, *p* < 0.001), lifetime violence (*β* = 0.037, *p* < 0.001), and IPV (*β* = 0.027, *p* < 0.001). This model explained 37.4% of the variance, *F*(8, 35,912) = 2,683, *R²adj* = 0.374, *p* < 0.001. The attenuation when adjusting for baseline depression is consistent with T1 depression partially mediating violence effects on T2 depression (see Fig. 3). Among psychosocial variables, limited social support emerged as the strongest protective factor (*β* = -0.066, *p* < 0.001), while greater loneliness predicted higher depression severity (*β* = 0.049, *p* < 0.001), highlighting the additive influence of social context on future mental health outcomes.

### Diagnostic Case-Control Analyses Using UK Biobank Clinical Depression Outcomes

Depression diagnosis versus no diagnosis pairs (*N* = 7,295 pairs; total *N* = 14,590) were matched on age (*range* = 40–70 years; *M* = 54.5, *SD* = 7.6) and sex (68% female). Detailed demographic characteristics of the two groups are presented in Table 1, and individual depressive symptom scores, violence exposure estimates, and psychosocial factors are summarised in Supplementary Tables 3–5. Individuals diagnosed with depression scored significantly higher on depression severity than those without a diagnosis at T1: *t*(11,209) = 41.91, *p* < 0.001, *d* = 0.74 and T2: *t*(11,398) = 42.9, *p* < 0.001, *d* = 0.71.

**Fig. 4:**
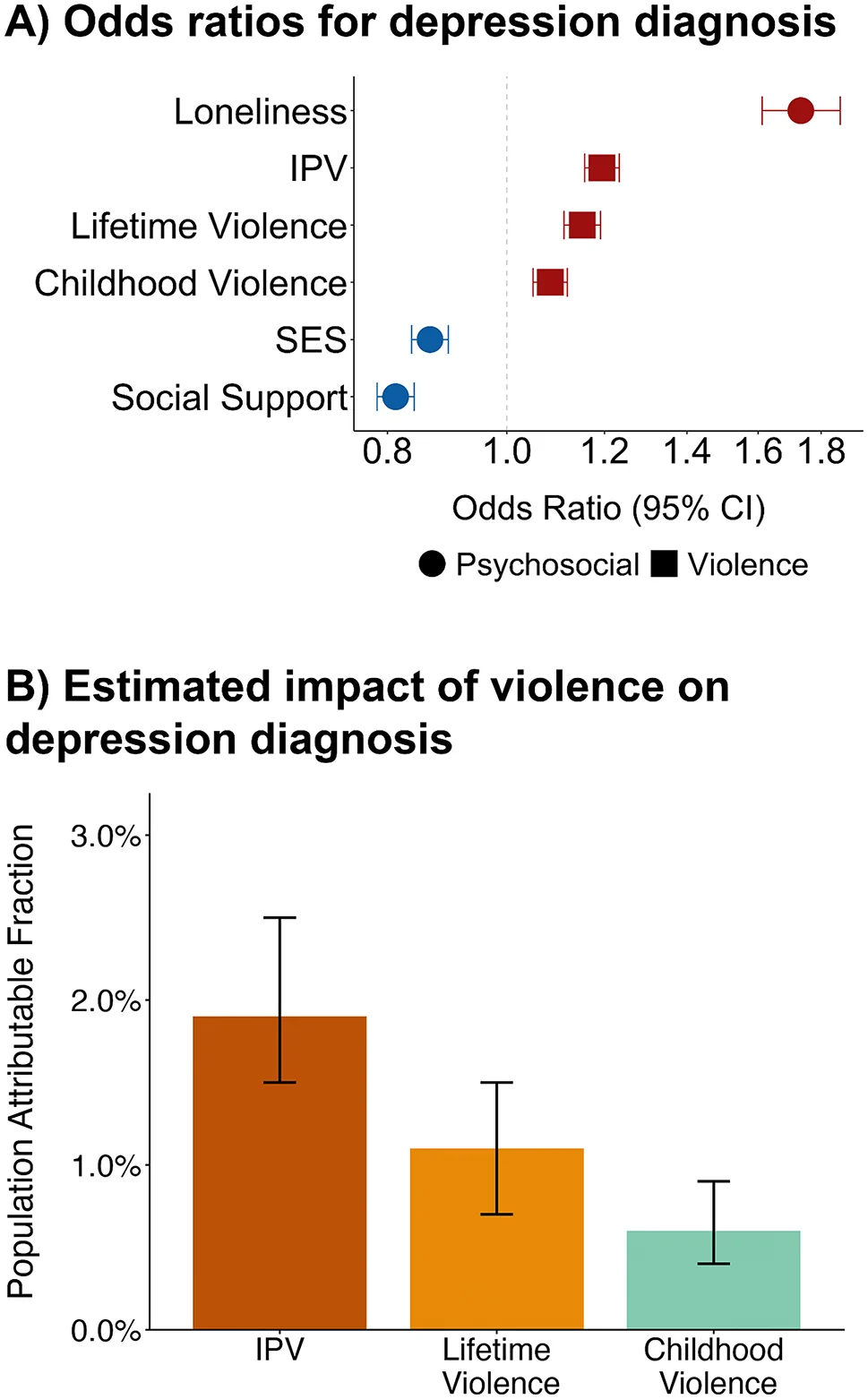
Violence exposures, psychosocial factors, and odds of clinical depression diagnosis. (**A**) Odds ratios and 95% confidence intervals from logistic regression models showing associations of loneliness, intimate partner violence (IPV), lifetime violence, childhood maltreatment, socioeconomic status (SES), and social support with depression diagnosis. (**B**) Population attributable fractions (PAFs) estimating the proportion of depression diagnoses attributable to different types of violence exposures. Error bars represent 95% confidence intervals.

In the initial logistic regression model adjusting for SES, all forms of violence were significantly associated with increased odds of a depression diagnosis: childhood maltreatment (OR = 1.08, 95% CI [1.05, 1.11]), lifetime violence (OR = 1.18, 95% CI [1.14, 1.22]), and IPV (OR = 1.17, 95% CI [1.14, 1.21]; see Fig. 4A). Sensitivity analyses additionally adjusting for parental history of depression yielded consistent results (childhood maltreatment OR = 1.07, 95% CI [1.04, 1.1]; lifetime violence OR = 1.17, 95% CI [1.13, 1.21]; IPV OR = 1.17, 95% CI [13, 1.21]). A second model adding social support and loneliness significantly improved model fit (*χ²* = 490.14, *p* < 0.001), increasing Nagelkerke *R*² from 0.029 to 0.08. All violence-related associations remained statistically significant. In this fully adjusted model, loneliness emerged as the strongest predictor of depression (OR = 1.73, 95% CI [1.61, 1.87]), followed by IPV (OR = 1.19, 95% CI [1.16, 1.23]), lifetime violence (OR = 1.15, 95% CI [1.11, 1.19]), and childhood maltreatment (OR = 1.08, 95% CI [1.05, 1.12], *p* < 0.001). Social support (OR = 0.81, 95% CI [0.78, 0.84]) was a significant protective factor. The final model demonstrated moderate discriminative ability (*AUC* = 0.646) and acceptable fit, although the Hosmer–Lemeshow test indicated some deviation from perfect model calibration (*χ²* = 45.18, *p* < 0.001).

Separate models for each violence type yielded modestly larger odds ratios compared to the simultaneous model: lifetime violence (OR = 1.24, 95% CI [1.2, 1.28]), IPV (OR = 1.23, 95% CI [1.19, 1.27]), and childhood maltreatment (OR = 1.15, 95% CI [1.11, 1.18]). This pattern mirrors the linear regression findings, indicating that simultaneous modelling produces conservative estimates of each violence type’s association with depression. Item-level analyses (Supplementary Table 6) revealed that IPV-related belittlement (OR = 1.34, 95% CI [1.28, 1.4]) and childhood ‘felt hated’ (OR = 1.19, 95% CI [1.14, 1.25]) showed the largest independent associations with depression diagnosis, while physical IPV was again the only non-significant predictor.

No significant interactions examining moderation by psychosocial variables were found, indicating that social support and loneliness likely exert independent effects on depression risk rather than moderating the impact of violence exposure.

Cumulative exposure models demonstrated a strong dose-response relationship. In the linear model, each additional type of violence exposure was associated with a 34.3% increase in odds of depression diagnosis (OR = 1.34, 95% CI [1.3, 1.39]). Categorical violence exposure was significantly associated with depression diagnosis, *χ²*(3) = 326.45, *p* < 0.001: compared to individuals with no violence exposure, those with one type had 36.7% higher odds (OR = 1.37, 95% CI [1.28, 1.47]), two types had 103% higher odds (OR = 2.03, 95% CI [1.89, 2.19]), and three types had 169% higher odds (OR = 2.69, 95% CI [2.37, 3.05]) of depression diagnosis. This model demonstrated the best fit (*AIC* = 19,310) compared to both the linear model (*AIC* = 19,352) and the model with individual violence types (*AIC* = 19,340). The nonlinear model, which included both linear and squared terms for cumulative violence, also improved fit (*AIC* = 19,341). The quadratic term was statistically significant (OR = 1.04, 95% CI [1.02, 1.06], *p* < 0.001), indicating that the risk of depression increased at an accelerating rate with higher cumulative violence exposure.

Based on the model adjusting for confounders, population attributable fractions (PAFs) were estimated at 0.6% for childhood maltreatment, 1.1% for lifetime violence, and 1.8% for IPV (see Fig. 4B). These findings suggest that IPV is associated with the greatest attributable burden to depression diagnoses in this sample, both in terms of effect size and prevalence.

## Discussion

This study systematically examined how violence during childhood, across the lifetime, and within intimate relationships contributes to depressive symptom severity and clinical depression diagnosis. We tested whether social support, SES and loneliness modified these associations and explored additive, interactive, and non-linear effects of cumulative violence exposure. Our findings provide robust evidence that all three forms of violence independently predict both depression severity and the likelihood of a clinical depression diagnosis. In cross-sectional analyses, lifetime violence emerged as the strongest predictor of depression severity, followed by IPV and childhood maltreatment. These associations remained significant but were attenuated after accounting for psychosocial factors, with loneliness emerging as the strongest independent psychosocial predictor. Importantly, our prospective analyses confirmed that violence exposure predicted future depression severity, even after controlling for initial depressive symptom levels, with consistent patterns observed across violence types. In matched case-control analyses of clinical depression, we observed similar patterns, with all three violence types significantly increasing the odds of depression diagnosis, an effect that persisted after adjustment for psychosocial factors.

The robust relationship between violence exposure and depression aligns with previous research identifying interpersonal violence as a significant risk factor for mood disorders [27, 28]. However, our findings extend beyond simple associations by elucidating complex patterns of risk with important implications for understanding the etiology of depression. Specifically, our analyses revealed that the relationship between violence and depression is not merely additive but also exhibits both interactive and non-linear properties. The observed synergistic interactions among different forms of violence suggest that experiencing multiple types of violence compounds psychological burden beyond the sum of their individual effects. This finding provides empirical support for theoretical frameworks that conceptualize polyvictimization as particularly detrimental to mental health [29, 30]. The presence of a significant quadratic term in our models further indicates that depression risk accelerates at higher levels of cumulative violence exposure. This pattern is consistent with stress sensitization theory, which posits that repeated exposure to adversity induces neurobiological changes that lower the threshold for stress reactivity, thereby amplifying vulnerability to subsequent stressors [31, 32]. Each additional violence exposure may compound this sensitization, explaining the observed nonlinear increase in depression risk. These findings extend previous research on cumulative childhood maltreatment to demonstrate similar patterns across the lifespan, underscoring the persistent vulnerability to revictimization among those with early violence exposure [33].

Notably, childhood maltreatment showed smaller independent effects than lifetime violence and IPV in both cross-sectional and prospective analyses. Several factors may account for this pattern. First, childhood maltreatment represents more temporally distant experiences compared to lifetime violence and IPV. Its effects may be partially indirect, operating through intervening life experiences including adult revictimization and relationship difficulties [9], which could explain why more recent violence exposures show stronger direct associations with depression [33]. Second, sensitive period effects may operate differently across violence types; while early adversity shapes developmental trajectories and stress reactivity, more proximal exposures such as IPV may exert stronger direct effects on current mood states. Third, retrospective reporting of childhood experiences may be subject to greater recall variability than reports of recent adult experiences. These considerations highlight the importance of examining violence across developmental periods rather than focusing on single exposure types.

Our results demonstrate that psychosocial factors moderate the association between violence exposure and depression, consistent with diathesis-stress and stress-buffering models of depression [10, 34]. Loneliness amplified violence-depression associations, while social support buffered this relationship across all violence types, with particularly pronounced protective effects for IPV. These findings replicate and extend earlier UK Biobank studies demonstrating that loneliness interacts with childhood maltreatment to heighten depression vulnerability [6], and that supportive relationships may mitigate psychological consequences of adversity [15]. Supportive relationships may be especially beneficial for mitigating the isolating effects of partner abuse [35]. From an intervention perspective, these findings suggest that addressing social isolation and strengthening social support networks may reduce depression risk among violence-exposed individuals, highlighting the potential utility of a transdiagnostic approach that addresses both violence-related mental health symptoms and broader aspects of psychosocial functioning [36].

The population attributable fraction (PAF) analyses provide valuable insights into the public health impact of different forms of violence. IPV demonstrated the highest PAF (1.8%), suggesting that almost one in 50 depression diagnoses in this population are associated with IPV exposure. While this estimate assumes a causal relationship and may appear modest, it represents a substantial burden of disease at the population level, highlighting IPV as a key prevention target. The relatively higher PAF for IPV compared to other violence types likely reflects both its strong association with depression and its prevalence within the sample. These findings suggest IPV, particularly emotional abuse, may be an important target for depression prevention efforts [37]. Notably, our item-level analyses indicated that emotional abuse (belittlement) showed the strongest association with depression, while physical IPV was not directly associated with depression. This specificity suggests that public health efforts focused on recognising and addressing emotional abuse within relationships, not only physical violence, may be relevant for depression prevention. However, these observational findings cannot establish causality, and intervention effectiveness would require direct evaluation.

Our prospective analyses, which demonstrated that violence exposure predicted future depressive symptoms even after controlling for initial symptom levels, have important implications for understanding depression chronicity. These findings indicate that violence exposure contributes not only to depression onset but also to its persistence and recurrence, potentially via biological mechanisms such as inflammatory processes, dysregulation of the hypothalamic-pituitary-adrenal axis, or epigenetic changes that endure long after the violence exposure [38, 39]. The modest effect sizes observed in our prospective analyses, coupled with the high stability of depressive symptoms over time, suggest that while violence exposure significantly influences depression trajectories, established depression also shows considerable continuity regardless of etiology.

Several methodological considerations inform interpretation of our findings. Among MHQ completers, participants with complete data on all study variables were similar to those excluded due to missing data on violence exposures, socioeconomic indicators, social support and loneliness. However, included participants had somewhat higher baseline depression, likely reflecting greater engagement with the MHQ among more symptomatic individuals. This selection toward a more symptomatic sample may limit generalisability to lower-severity populations, though it may also have increased statistical power to detect violence-depression associations. Additionally, UK Biobank participants are healthier, wealthier, and more educated than the general UK population [18], which may further limit generalisability and potentially underestimate violence exposure prevalence and effect sizes.

Several limitations should be acknowledged when interpreting these findings. First, our violence measures, while comprehensive, relied on retrospective self-report, which may be subject to recall bias or influenced by current mood states [40]. Second, although our longitudinal design allowed us to establish temporal precedence, the observational nature of the study precludes definitive causal inferences. Third, we lacked measures of childhood SES (e.g., parental income or education during childhood), which may confound associations between childhood maltreatment and later depression. Fourth, causal ordering among study variables warrants consideration. IPV may partially mediate associations between childhood maltreatment and depression. Simultaneous modelling of all violence types may therefore underestimate total effects of childhood maltreatment. We addressed this by reporting both separate and simultaneous models. Similarly, loneliness and social support may lie on the causal pathway between violence and depression. Primary effect estimates were therefore derived from models adjusting for confounders only. Fifth, loneliness was assessed with a single item, which may limit measurement reliability compared to multi-item scales. Finally, although we examined several key psychosocial moderators, other potentially important factors such as coping styles, personality traits, or genetic susceptibility were not assessed. Future research would benefit from integrating biological data (e.g., inflammatory markers, neuroimaging, genetic profiles) with psychosocial measures to build more comprehensive biopsychosocial models of depression vulnerability following violence exposure. Our matched case-control sample was predominantly female. While all models controlled for sex, we did not conduct sex-stratified analyses. Given that IPV is more commonly experienced by women and may have sex-specific psychological consequences, future research should examine whether violence-depression associations differ by sex. Additionally, person-centered approaches that identify distinct trajectories of resilience and vulnerability following violence exposure may help to inform the development of more tailored and effective intervention strategies.

In conclusion, this study demonstrates that violence exposure across the lifespan contributes to depression risk through cumulative, interactive, and non-linear mechanisms. The identification of loneliness as a robust psychosocial risk factor, and social support as a protective buffer, highlights modifiable intervention targets that could mitigate the mental health burden associated with violent experiences. Collectively, these findings emphasize the importance of assessing multiple forms of violence in the clinical evaluation of depression and underscore the need for prevention and intervention strategies that enhance social connection alongside addressing violence-related mental health symptoms.

## Supplementary Information

Below is the link to the electronic supplementary material.

Supplementary Material 1

## Data Availability

No datasets were generated or analysed during the current study.
