# Supplementary material for: Violence across the lifespan and its association with depression: cumulative effects and psychosocial moderation in the UK Biobank
Source: Soc Psychiatry Psychiatr Epidemiol. 2026 Apr 29;61(9):1769–80. doi: 10.1007/s00127-026-03079-3 (PMC13578047; doi:10.1007/s00127-026-03079-3)
Supplement: Supplementary file 1 [file 127_2026_3079_MOESM1_ESM.docx]

| **Supplementary Table 1.** UK Biobank Variables Reference Table | | | | | |
| --- | --- | --- | --- | --- | --- |
| **Measure** | **Field ID** | **Answer Options** | **Original coding** | **New coding** | **Composite index** |
| ***Depressive Symptoms (PHQ-9 Items) T1*** | | | | | |
| Movement | 20518 | Not at all  Several Days  More than half the days  Nearly every day  Prefer not to answer | 1  2  3  4  -818 | 0  1  2  3  NA | Sum of all 9 items |
| Feeling depressed | 20510 |  |  |  |  |
| Feelings of inadequacy | 20507 |  |  |  |  |
| Fatigue | 20519 |  |  |  |  |
| Anhedonia | 20514 |  |  |  |  |
| Appetite problems | 20511 |  |  |  |  |
| Suicidal thoughts | 20513 |  |  |  |  |
| Concentration difficulties | 20508 |  |  |  |  |
| Sleep problems | 20517 |  |  |  |  |
| ***Depressive Symptoms - Follow-up (PHQ-9 Items) T2*** | | | | | |
| Movement | 29009 | Not at all  Several Days  More than half the days  Nearly every day  Prefer not to answer | 0  1  2  3  -3 | 0  1  2  3  NA | Sum of all 9 items |
| Feeling depressed | 29003 |  |  |  |  |
| Feelings of inadequacy | 29007 |  |  |  |  |
| Fatigue | 29005 |  |  |  |  |
| Anhedonia | 29002 |  |  |  |  |
| Appetite problems | 29006 |  |  |  |  |
| Suicidal thoughts | 29010 |  |  |  |  |
| Concentration difficulties | 29008 |  |  |  |  |
| Sleep problems | 29004 |  |  |  |  |
| ***Depression Diagnosis*** | | | | | |
| Depression ICD9 | 41271 | Neurotic depression (dysthymia) | 3004 | 1 | Depression diagnosis assigned if 1 on one or more codes |
|  |  | Depressive Disorder not elsewhere classified | 3119 | 1 |  |
| Depression ICD10 | 41270 | Depressive episode | F32 | 1 |  |
|  |  | Mild depressive episode | F320 | 1 |  |
|  |  | Moderate depressive episode | F321 | 1 |  |
|  |  | Severe depressive episode without psychotic symptoms | F322 | 1 |  |
|  |  | Severe depressive episode with psychotic symptoms | F323 | 1 |  |
|  |  | Other depressive episodes | F328 | 1 |  |
|  |  | Depressive episode, unspecified | F329 | 1 |  |
|  |  | Recurrent depressive disorder | F33 | 1 |  |
|  |  | Recurrent depressive disorder, current episode mild | F330 | 1 |  |
| Mental Health diagnosis | 29000 | Depression | 33988 | 1 |  |
| ***Childhood Maltreatment T1*** | | | | | |
| Childhood physical abuse | 20488 | Never true  Rarely true  Sometimes true  Often  Very often true  Prefer not to answer | 0  1  2  3  4  -818 | 0  1  2  3  4  NA | Standardized and summed individual items to composite childhood maltreatment index |
| Childhood sexual abuse | 20490 |  |  |  |  |
| Felt loved as a child (reverse scored) | 20489 |  |  |  |  |
| Felt hated as a child | 20487 |  |  |  |  |
| ***Lifetime Violence T1*** | | | | | |
| Sexual assault | 20531 | Never  Yes, but not in the last 12 months  Yes, within the last 12 months  Prefer not to answer | 0  1  2  -818 | 0  1  2  NA | Standardized and summed individual items to composite lifetime violence index |
| Violent crime victim | 20529 |  |  |  |  |
| Violent death witness | 20530 |  |  |  |  |
| ***Intimate Partner Violence (IPV) T1*** | | | | | |
| Confiding (reverse scored) | 20522 | Never true  Rarely true  Sometimes true  Often  Very often true  Prefer not to answer | 0  1  2  3  4  -818 | 0  1  2  3  4  NA | Standardized and summed individual items to composite IPV index |
| Physical violence | 20523 |  |  |  |  |
| Belittlement | 20521 |  |  |  |  |
| Sexual violence | 20524 |  |  |  |  |
| ***Socio-economic status (SES)*** | | | | | |
| Income level | 738 | Less than 18,000  18,000 to 30,999  31,000 to 51,999  52,000 to 100,000  Greater than 100,000  Prefer not to answer  Do not know | 1  2  3  4  5  -3  -1 | 1  2  3  4  5  NA  NA | Standardized and summed items to composite SES index |
| Highest educational qualification | 6138 | Prefer not to answer  College or University degree  A levels/AS levels or equivalent  O levels/GCSEs or equivalent  CSEs or equivalent  NVQ or HND or HNC or equivalent  Other professional qualifications  None of the above | -3  1  2  3  4  5  6  -7 | NA  6  5  4  3  2  1  NA |  |
| ***Social Support*** | | | | | |
| Friendships satisfaction | 4570 | Extremely happy  Very happy  Moderately happy  Moderately unhappy  Very unhappy  Extremely unhappy  Do not know  Prefer not to answer | 1  2  3  4  5  6  -1  -3 | 6  5  4  3  2  1  NA  NA | Standardized and summed items to composite social support index |
| Family satisfaction | 4559 | Extremely happy  Very happy  Moderately happy  Moderately unhappy  Very unhappy  Extremely unhappy  Do not know  Prefer not to answer | 1  2  3  4  5  6  -1  -3 | 6  5  4  3  2  1  NA  NA |  |
| Social activities | 6160 | Sports club or gym  Pub or social club  Religious group  Adult education class  Other group activity  None of the above  Prefer not to answer | 1  2  3  4  5  -7  -3 | Sum of activities selected |  |
|  |  |  |  | NA |  |
|  |  |  |  | NA |  |
| Ability to confide in someone | 2110 | Almost daily  2-4 times a week  About once a week  About once a month  Once every few months  Never or almost never  Do not know  Prefer not to answer | 5  4  3  2  1  0  -1  -3 | 5  4  3  2  1  0  NA  NA |  |
| ***Loneliness*** | | | | | |
| Loneliness, isolation | 2020 | Yes  No  Do not know  Prefer not to answer | 1  0  -1  -3 | 1  0  NA  NA | Standardized |
| ***Covariates*** |  |  |  |  |  |
| Age | 21022 | Age at recruitment |  |  |  |
| Sex | 31 | Female  Male | 0  1 | 0  1 |  |

*Note.* NA = not applicable, PHQ-9 = Patient Health Questionnaire-9; T1 = Time 1; T2 = Time 2.

| **Supplementary Table 2.** Comparison of Included and Excluded Participants Among Mental Health Questionnaire Completers | | | | |
| --- | --- | --- | --- | --- |
| **Variable** | **Included**  (N = 35,921) | **Excluded**  (N = 136,945) | ***p*** | **Cohen's *d*** |
| Age | 56.05 (7.57) | 55.24 (7.53) | < 0.001 | 0.11 |
| Female (%) | 56.1% | 58.1% | < 0.001 |  |
| PHQ-9 (T1) | 2.65 (3.57) | 1.52 (3.02) | < 0.001 | 0.34 |
| Income | 2.99 (1.16) | 2.93 (1.15) | < 0.001 | 0.05 |
| Education | 1.69 (1.20) | 1.76 (1.29) | < 0.001 | -0.06 |
| Childhood physical abuse | 0.30 (0.68) | 0.29 (0.67) | 0.004 | 0.02 |
| Childhood sexual abuse | 0.15 (0.52) | 0.14 (0.52) | 0.333 | 0.01 |
| Felt loved (reverse) | 0.77 (0.98) | 0.77 (0.97) | 0.632 | 0.00 |
| Felt hated | 0.30 (0.77) | 0.29 (0.76) | 0.012 | 0.02 |
| Sexual assault | 0.16 (0.37) | 0.16 (0.36) | 0.145 | 0.01 |
| Violent crime | 0.21 (0.42) | 0.19 (0.41) | < 0.001 | 0.03 |
| Witnessed violent death | 0.14 (0.37) | 0.14 (0.36) | 0.032 | 0.01 |
| Confiding (reverse) | 1.01 (1.27) | 1.00 (1.27) | 0.092 | 0.01 |
| Physical IPV | 0.22 (0.66) | 0.22 (0.64) | 0.125 | 0.01 |
| Belittlement | 0.45 (0.92) | 0.45 (0.91) | 0.401 | 0.01 |
| Sexual IPV | 0.10 (0.43) | 0.09 (0.43) | 0.268 | 0.01 |
| Loneliness | 0.12 (0.47) | 0.12 (0.51) | 0.007 | 0.02 |

Notes. Included participants had complete data on all study variables. Results are reported as mean (standard deviation) or percentage (%) where indicated. Group differences were tested using independent samples t-tests (Welch's) for continuous variables and chi-square tests for categorical variables. Social support items (satisfaction with friendships, satisfaction with family relationships) could not be compared across groups as these variables were the primary source of missing data among MHQ completers (N = 110,433 missing). Cohen's *d*: 0.2 = small, 0.5 = medium, 0.8 = large.

| **Supplementary Table 3.** Depressive symptom scores | | | |
| --- | --- | --- | --- |
| **Variable** | **Total Sample**  **(N = 36,378)** | **Depression Group**  **(N = 7,295)** | **Matched Controls**  **(N = 7,295)** |
| PHQ-9 Item 1: Movement (T1) | 0.07 (0.32) | 0.18 (0.51) | 0.04 (0.23) |
| PHQ-9 Item 2: Feeling depressed (T1) | 0.26 (0.55) | 0.55 (0.77) | 0.19 (0.46) |
| PHQ-9 Item 3: Feelings of inadequacy (T1) | 0.25 (0.59) | 0.56 (0.85) | 0.18 (0.48) |
| PHQ-9 Item 4: Fatigue (T1) | 0.64 (0.79) | 1.03 (0.96) | 0.56 (0.72) |
| PHQ-9 Item 5: Anhedonia (T1) | 0.23 (0.55) | 0.49 (0.78) | 0.16 (0.44) |
| PHQ-9 Item 6: Appetite problems (T1) | 0.25 (0.62) | 0.54 (0.88) | 0.20 (0.52) |
| PHQ-9 Item 7: Suicidal thoughts (T1) | 0.05 (0.27) | 0.15 (0.46) | 0.02 (0.18) |
| PHQ-9 Item 8: Concentration difficulties (T1) | 0.22 (0.54) | 0.47 (0.78) | 0.16 (0.45) |
| PHQ-9 Item 9: Sleep problems (T1) | 0.70 (0.89) | 1.04 (1.01) | 0.64 (0.84) |
| PHQ-9 Total Score (T1) | 2.66 (3.58) | 5.00 (5.12) | 2.15 (2.76) |
| PHQ-9 Item 1: Movement (T2) | 0.07 (0.33) | 0.17 (0.53) | 0.04 (0.25) |
| PHQ-9 Item 2: Feeling depressed (T2) | 0.28 (0.58) | 0.60 (0.81) | 0.21 (0.48) |
| PHQ-9 Item 3: Feelings of inadequacy (T2) | 0.24 (0.59) | 0.55 (0.86) | 0.17 (0.49) |
| PHQ-9 Item 4: Fatigue (T2) | 0.65 (0.83) | 1.09 (1.00) | 0.56 (0.75) |
| PHQ-9 Item 5: Anhedonia (T2) | 0.26 (0.59) | 0.58 (0.85) | 0.18 (0.48) |
| PHQ-9 Item 6: Appetite problems (T2) | 0.24 (0.62) | 0.54 (0.88) | 0.18 (0.52) |
| PHQ-9 Item 7: Suicidal thoughts (T2) | 0.05 (0.28) | 0.15 (0.48) | 0.03 (0.20) |
| PHQ-9 Item 8: Concentration difficulties (T2) | 0.24 (0.57) | 0.52 (0.82) | 0.18 (0.49) |
| PHQ-9 Item 9: Sleep problems (T2) | 0.71 (0.91) | 1.06 (1.05) | 0.66 (0.86) |
| PHQ-9 Total Score (T2) | 2.74 (3.73) | 5.25 (5.30) | 2.20 (2.94) |

*Notes.* PHQ-9 = Patient Health Questionnaire-9; Values are presented as Mean (standard deviation); T1 = Time 1; T2 = Time 2.

| **Supplementary Table 4.** Violence Exposure | | | |
| --- | --- | --- | --- |
| **Variable** | **Total Sample**  **(N = 36,378)** | **Depression Group**  **(N = 7,295)** | **Matched Controls**  **(N = 7,295)** |
| Childhood maltreatment |  |  |  |
| Childhood physical abuse | 0.30 (0.69) | 0.44 (0.86) | 0.28 (0.64) |
| Childhood sexual abuse | 0.15 (0.53) | 0.26 (0.72) | 0.13 (0.48) |
| Felt loved as a child (reverse scored) | 0.78 (0.98) | 1.07 (1.12) | 0.70 (0.92) |
| Felt hated as a child | 0.30 (0.78) | 0.57 (1.06) | 0.25 (0.68) |
| Lifetime violence |  |  |  |
| Sexual assault | 0.16 (0.37) | 0.25 (0.44) | 0.16 (0.37) |
| Violent crime victim | 0.21 (0.42) | 0.24 (0.44) | 0.19 (0.40) |
| Violent death witness | 0.14 (0.37) | 0.15 (0.38) | 0.12 (0.34) |
| Intimate Partner Violence (IPV) |  |  |  |
| Confiding (reverse scored) | 1.02 (1.28) | 1.17 (1.28) | 0.98 (1.28) |
| Physical violence | 0.22 (0.66) | 0.38 (0.87) | 0.21 (0.64) |
| Belittlement | 0.45 (0.92) | 0.81 (1.19) | 0.41 (0.87) |
| Sexual violence | 0.10 (0.43) | 0.19 (0.61) | 0.10 (0.43) |

*Notes.* Values are presented as Mean (standard deviation); composite scores are standardized (z-scores).

| **Supplementary Table 5.** Psychosocial Factors | | | |
| --- | --- | --- | --- |
| **Variable** | **Total Sample**  **(N = 36,378)** | **Depression Group**  **(N = 7,295)** | **Matched Controls**  **(N = 7,295)** |
| Socioeconomic status (SES) |  |  |  |
| Income level | 2.99 (1.16) | 2.81 (1.17) | 3.08 (1.15) |
| Highest educational qualification | 1.69 (1.20) | 1.72 (1.19) | 1.69 (1.17) |
| Social Support |  |  |  |
| Friendships satisfaction | 4.70 (0.84) | 4.53 (0.94) | 4.76 (0.80) |
| Family satisfaction | 4.75 (0.95) | 4.48 (1.06) | 4.81 (0.90) |
| Social activities | 1.07 (0.88) | 1.03 (0.89) | 1.08 (0.87) |
| Ability to confide in someone | 4.57 (1.86) | 4.48 (1.84) | 4.67 (1.80) |
| Loneliness | 0.12 (0.47) | 0.26 (0.60) | 0.09 (0.42) |

*Notes.* Values are presented as Mean (standard deviation); composite scores are standardized (z-scores).

| **Supplementary Table 6.** Independent Associations of Individual Maltreatment and Violence Items with Depression Severity (Linear Regression) and Depression Diagnosis (Logistic Regression) | | | |
| --- | --- | --- | --- |
|  | **Linear Regression** | | **Logistic Regression** |
| **Variable** | ***β*** | ***p*** | **OR [95% CI]** |
| Childhood physical abuse | 0.022 | < 0.001 | 1.0 [0.96, 1.04] |
| Childhood sexual abuse | 0.035 | < 0.001 | 1.08 [1.04, 1.13] |
| Felt loved as a child (reverse scored) | -0.075 | < 0.001 | 0.85 [0.81, 0.88] |
| Felt hated as a child | 0.083 | < 0.001 | 1.19 [1.14, 1.25] |
| Sexual assault | 0.028 | < 0.001 | 1.09 [1.05, 1.14] |
| Violent crime victim | 0.017 | < 0.001 | 1.04 [1.0, 1.08] |
| Violent death witness | 0.026 | < 0.001 | 1.03 [1.0, 1.07] |
| Confiding (reverse scored) | -0.038 | < 0.001 | 1.0 [0.97, 1.04] |
| Physical violence | -0.004 | 0.538 | 1.0 [0.96, 1.05] |
| Belittlement | 0.144 | < 0.001 | 1.34 [1.28, 1.4] |
| Sexual violence | 0.012 | 0.038 | 0.95 [0.91, 0.99] |

Notes. *β* = standardized regression coefficient from linear regression predicting depression severity (PHQ-9 scores) in the full sample (N = 35,921). OR = odds ratio from logistic regression predicting depression diagnosis in the matched case-control sample (N = 14,590). All models adjusted for socioeconomic status, social support, and loneliness, age, age², and sex. Individual items were z-scored prior to entry.

**Supplementary Figure 1.** Interaction effects between violence types on depression severity.

Predicted depression severity (PHQ-9 scores) as a function of violence exposure, stratified by levels of co-occurring violence type (±1 SD from the mean). Diverging lines indicate synergistic effects: the association between one violence type and depression is stronger at higher levels of the other violence type.
